# The Role of Immunity in the Pathogenesis of SARS-CoV-2 Infection and in the Protection Generated by COVID-19 Vaccines in Different Age Groups

**DOI:** 10.3390/pathogens12020329

**Published:** 2023-02-15

**Authors:** Zainalabideen A. Abdulla, Sharaf M. Al-Bashir, Hiba Alzoubi, Noor S. Al-Salih, Ala A. Aldamen, Ahmed Z. Abdulazeez

**Affiliations:** 1 Department of Clinical Sciences, Faculty of Medicine, Yarmouk University, Irbid 21163, Jordan; 2 Department of Basic Medical Sciences, Faculty of Medicine, Yarmouk University, Irbid 21163, Jordan; 3 Internship Program, Princess Basma Teaching Hospital, Irbid 26125, Jordan

**Keywords:** SARS-CoV-2, COVID-19, vaccines, immune response, children vaccination, multisystem inflammatory syndrome in children (MIS-C)

## Abstract

This study aims to review the available data regarding the central role of immunity in combating SARS-CoV-2 infection and in the generation of protection by vaccination against COVID-19 in different age groups. Physiologically, the immune response and the components involved in it are variable, both functionally and quantitatively, in neonates, infants, children, adolescents, and adults. These immunological differences are mirrored during COVID-19 infection and in the post-vaccination period. The outcome of SARS-CoV-2 infection is greatly dependent on the reaction orchestrated by the immune system. This is clearly obvious in relation to the clinical status of COVID-19 infection, which can be symptomless, mild, moderate, or severe. Even the complications of the disease show a proportional pattern in relation to the immune response. On the contrary, the commonly used anti-COVID-19 vaccines generate protective humoral and cellular immunity. The magnitude of this immunity and the components involved in it are discussed in detail. Furthermore, many of the adverse effects of these vaccines can be explained on the basis of immune reactions against the different components of the vaccines. Regarding the appropriate choice of vaccine for different age groups, many factors have to be considered. This is a cornerstone, particularly in the following age groups: 1 day to 5 years, 6 to 11 years, and 12 to 17 years. Many factors are involved in deciding the route, doses, and schedule of vaccination for children. Another important issue in this dilemma is the hesitancy of families in making the decision about whether to vaccinate their children. Added to these difficulties is the choice by health authorities and governments concerning whether to make children’s vaccination compulsory. In this respect, although rare and limited, adverse effects of vaccines in children have been detected, some of which, unfortunately, have been serious or even fatal. However, to achieve comprehensive control over COVID-19 in communities, both children and adults have to be vaccinated, as the former group represents a reservoir for viral transmission. The understanding of the various immunological mechanisms involved in SARS-CoV-2 infection and in the preparation and application of its vaccines has given the sciences a great opportunity to further deepen and expand immunological knowledge. This will hopefully be reflected positively on other diseases through gaining an immunological background that may aid in diagnosis and therapy. Humanity is still in continuous conflict with SARS-CoV-2 infection and will be for a while, but the future is expected to be in favor of the prevention and control of this disease.

## 1. Introduction

In the USA, as well as throughout the world, fewer cases of COVID-19 have been reported in children than in adults. They represent approximately 17–18% of all COVID-19 cases among the population [1]. The percentage of hospitalized children is between 1.7% and 4.4% of total hospitalizations [2]; however, up to 50% of infected children and adolescents have COVID-19 with no symptoms [3]. This can shed light on the competency of the immune system in combating the SARS-CoV-2 virus in children in comparison to adults. For these reasons, a question is raised: “Why do we vaccinate children?” The answer to this question is to prevent severe disease and the spread of infection to families and communities. Furthermore, rarely, neonates may be at a higher risk of getting severe COVID-19 than older children [3]. This is because the immune system in infants is still immature in comparison to older children [3].

When a person is born, both innate and passive immunity are transferred to the newborn baby from the mother via the placenta and during breastfeeding, which can protect the baby for a while until their adaptive immunity matures [4]. By the age of four years, the immune system tends to be more responsive with good memory cell generation. By the age of 55 years, the immune system has lost some of its responsive power and memory to fight infections. Booster doses of vaccines can help to potentiate the immune response [4].

SARS-CoV-2 can infect all age groups, and there are striking differences in the immune response and outcome of COVID-19 infection between these groups. Neonates, infants, children, and adolescents commonly contract asymptomatic or mild COVID-19 [5,6,7]. In these age groups, the immune system works differently than in adults. The immune response in neonates is organized on the basis of immunological tolerance, rather than on resistance to microbes [8,9]. Basically, the immune response is regulated to prevent excessive damage to normal tissues [10].

In neonates/infants, the number of immunosuppressive cells is increased to reduce unwanted immune responses, and the role of these cells can determine the pattern of COVID-19 infection outcomes in babies [11]. During COVID-19 infection, the production of proinflammatory cytokines (IL-1ß, IL-6, IL-8, IL-17, and TNF-α) is also more tightly regulated in neonates, infants, and children in comparison to adults [12].

The effect of the SARS-CoV-2 virus on the expression and function of angiotensin-converting enzyme 2 (ACE2) in children is different from that of adults and can determine the outcome of COVID-19 infection [5,13,14]. The SARS-CoV-2 virus gains entry to the body’s cells via the ACE2 receptor, and the virus can also reduce the expression of ACE2 receptors on many organs (alveolar cells, nasal mucosa, upper respiratory tract, endothelium, heart, kidney, and gut cells) that normally show them [5,13,14]. This can result in the accumulation of ACE2 in the respiratory tract, leading to lung injury [5]. Infants and children who show high levels of plasma ACE2 may be spared from this effect, as their ACE2 tissue expression is different from that of adults [15]. Moreover, ACE2 expression is also directly related to the androgen and estrogen levels of individuals, which decline with age [16].

Several weeks after the onset of COVID-19 symptoms, children may develop a serious complication: multisystem inflammatory syndrome (MIS; see below, Section 4.3.5). Children appear to have a lesser role in the spread of SARS-CoV-2 than they do in the transmission of other microorganisms. This related factor should be addressed when determining the benefits of immunizing children against COVID-19, as fewer than 2% of diagnosed cases include youngsters [17,18,19]. Children aged under five years are much less vulnerable to the coronavirus than adults, but they are also not invulnerable to the serious consequences. Therefore, if COVID-19 infection needs to be prevented, the vaccination of children is a pragmatic approach for this purpose [20].

The main aims of this review were, first, to show the differences in the immune response against SARS-CoV-2 in different age groups, from infancy to adolescence, in comparison to adults; second, we looked at the outcomes and complications of COVID-19 among these different age groups in relation to the immunological status. For these purposes, this review is organized into four major categories (Section 2, Section 3, Section 4 and Section 5), which are discussed below.

## 2. Review of the Literature and Discussion

### 2.1. Development and Maturation of the Normal Immune System

The immune system (IMS) evolves from a state of immaturity at birth to a state of maturity during adulthood and then declines during old age (immunosenescence). This maturation process passes into different phases that can be categorized into three age-related stages, which are the basis for understanding the immune response during COVID-19 infection and post-vaccination.

#### 2.1.1. The First Stage during Infancy

##### Innate Immunity

The functions of the different components of innate immunity are weak during infancy in comparison to in the later age groups. Infants have poor neutrophil function, making them susceptible to both viral and bacterial infections, which may reflect the low capability of neutrophil and endothelial adhesive functions (Table 1) [21,22,23]. 

The monocyte–macrophage series also shows reduced functions and poor secretion of bioactive molecules at birth. Although the number of pulmonary macrophages is reduced, this is corrected within a few days. Furthermore, the cord blood contains fewer myeloid dendritic cells (mDCs) than the peripheral blood of children and adults. These cells express fewer costimulatory molecules (HLA-II, CD80, and CD86) than an adult’s mDCs [29]. Again, these cells produce less IL-12, which links innate immunity with adaptive immunity. Therefore, the priming of Th1 and CD8 cells is affected [31]. Meanwhile, these cells respond to Toll-like receptor 4 (TLR4) stimulation and, thus, promote the function of Th17 cells via proinflammatory cytokine secretion (Table 1).

Natural killer (NK) cells, which are a component of the innate immune system, are less susceptible to cytokine activation by IL-12 and IL-15 and release less gamma interferon, resulting in a decreased antiviral response [32]. In addition, in the innate arm of newborns, NK cells have functional deficits, including decreased cell adhesion due to a decrease in selectins, decreased TNF production, and an increase in inhibitory receptors. The number of neonatal PDCs and the amount of type I IFN generated by these cells are reduced [60]. The main differences between NK cells and mDCs are based on the concept that the former cells are engaged with autologous infective or defective cells in a non-HLA restricted pattern, while the latter cells are engaged with cells of different organisms, such as viral pathogens [29,45]. 

Lastly, the alternative complement pathway and the lectin-binding activation pathway components, which are also innate immunity elements, are significantly present in lower concentrations than an adult’s level, leading to decreases in complement activities, such as opsonization, lysis of the target cells, and mediation of inflammatory processes against microbial molecules [33]. Therefore, innate immunity for protective activities is severely compromised after birth.

##### Adaptive Immunity

Adaptive immunity is also different during infancy compared with adulthood. These differences involve both T and B cells. After birth, peripheral Treg cells (Fox 3-positive) persist for an extended period [36], at a concentration that is approximately 3% of the total CD4+ T cells population [35], to mediate the anti-inflammatory response [37]. This can lead to reduced recognition of allo-antigens, a poor response to foreign antigens, and a skewed T cell response toward Th2 immunity. However, to compensate for the immaturity of Th1 cell functions, T cells with gamma–delta T cell receptors (γδ-TCRs) can produce significant amounts of gamma-interferon after brief polyclonal stimulation during neonatal infections [39,40].

On the contrary, as a humoral immunity responder, B cells are of two types. First, there are B2 cells, which secrete low-affinity IgM against limited antigens, including bacterial polysaccharides, and produce IL-10 and transforming growth factor (TGF). The latter two factors can promote the Th2 response [42]. B1 cells (conventional cells) have a broad repertoire of immunoglobulin responses in secondary lymphoid organs and bone marrow and comprise approximately 40% of the peripheral blood’s B cells from birth to a few months afterwards [61].

In addition, adaptive immunity responses to T cell-dependent antigens in neonates are significantly impaired in comparison to those of older children and adults [43]. This information is relevant for vaccination programs involving neonates and infants. Therefore, due to the impaired innate immunity and weak Th1 and antibody responses in neonates, the mortality rate among neonates from exposure to microbes other than SARS-CoV-2 is high [49].

**Figure 1:**
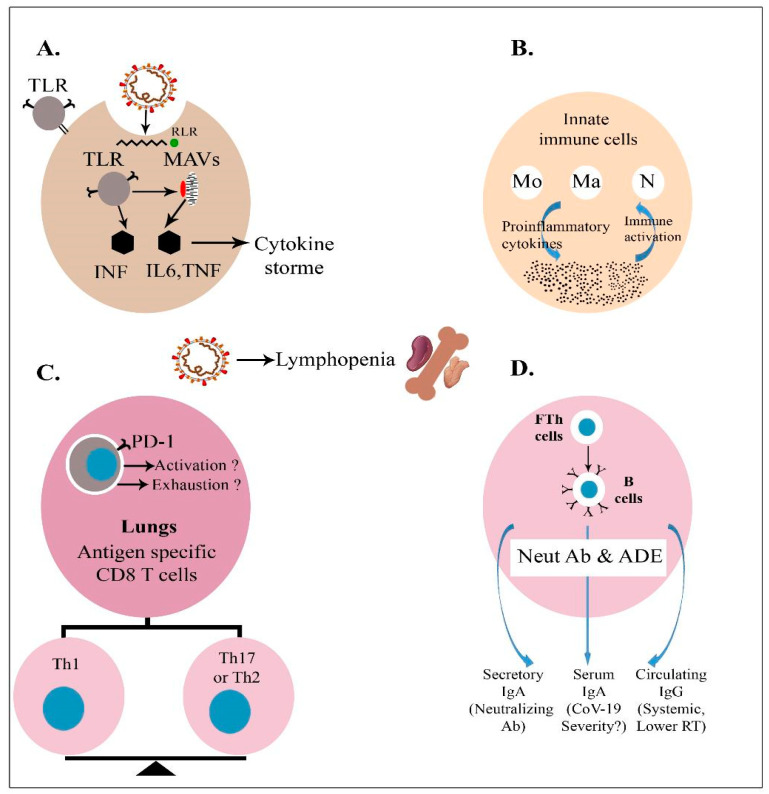
The roles of innate immunity (**A**,**B**) and adaptive immunity (**C**,**D**) in protection against SARS-CoV-2 infection. TLRs, Toll-like receptors; RLR, retinoic acid-inducible gene-1-like receptor; MAVS, mitochondrial antiviral signaling; INF, interferon; Mo, monocyte; Ma, macrophage; N, neutrophil; PD-1, programmed cell death protein; Neut Ab, neutralizing antibody; ADE, antibody-dependent enhancement; IL, interleukin.

Finally, potent adaptive immune responses depend on the potential of DCs to undergo maturation alongside the robust activation, differentiation, and survival of T and B cells. Newborn DCs have a diminished capacity to respond to pattern-associated molecular patterns (PAMPs) via Toll-like receptors (TLRs) and retinoic acid-inducible gene-I-like (RIG-I) receptors, resulting in the impaired expression of costimulatory molecules (CD40, CD80 and CD86) and decreased production of IL-12p70, which is essential for the differentiation of CD4 Th1 cells and CD8+ T cells. Newborn DCs also exhibit enhanced IL-4 production. CD4+ T cell differentiation is skewed toward the production of Th2 cells due to the low concentration of IL-12p70 and the increased production of IL-4. The reduced IL-12p70 concentration has an effect on CD8+ T cell development, resulting in lower IFN production (Figure 1). Neonates and infants also exhibit enhanced Treg responses. There are also functional abnormalities in newborn T follicular helper (Tfh) cells, including limited growth, which results in inadequate germinal center responses and decreased generation of high-affinity and isotype-switched antibodies [60].

#### 2.1.2. The Second Stage: From Childhood to Adolescence–Adulthood

In children, both innate and adaptive immunity start to mature compared with their levels in infants, but they still suffer from significant microbial infections. In addition, the concentration of maternal immunoglobulin transmitted via the placenta and through breastfeeding starts to wane by the age of six months [50,51].

During childhood, the number of Treg cells declines, while the number of primed memory Th1, Th2, and Th17 cells increases to be proportional to that of CD45R-positive naïve T cells [48]. This physiological maturation in the immune-competent cell population can provide the body with different armories to combat infections through cross-reactions between the epitopes of microbes and the body’s repertoire of memory T cells, even those that the body has not been exposed to previously [53,56,57].

B cells show similar patterns of cross-reactivity and antibody production against microbial antigens, such as the stimulation of natural isohemagglutinin (anti-ABO) of the IgM class by the intestinal flora [62].

Clinical/symptomatic and subclinical/asymptomatic infections can provoke antibody formation and the priming of T cells [44,63]. Viral infections and intracellular bacterial infections can provoke strong CD4 and CD8 T cell responses [64]. Sometimes, microbial superantigens can cause excessive stimulation of these cells, leading to the deletion of certain clones [44,63,64].

B cells go into somatic hypermutation, particularly at the hot spots of variable regions of immunoglobulin [49,65,66]. This mechanism is not required for T cells, as there is no need for high-affinity T cell receptors, since low-affinity T cell activation is enhanced by the actions of costimulatory coreceptors [66]. Added to this, HLA genetic polymorphism [67] and the genes controlling innate immunity can all shed light on the complexity of the adult immune response [44,63].

During an immune response, plasma cells that produce antibodies travel to the bone marrow, where they reside for various amounts of time. In addition, there may be constant regeneration of memory B cells in the presence of persistent antigen and T helper cells. Particulate antigens are retained by follicular dendritic cells for years in the lymph nodes [44]. Antigen persistence and cross-reactive antigens likely contribute to the survival of these B cells, which divide and secrete antibodies [44,61,62,63].

In conclusion, the immune system copes with the changes from fetal to infantile life. Then, it matures and extends during adulthood and later decreases during immunosenescence. Memory cells expand in the body over the lifetime. The immune system of senesced individuals is proportional to that of a newborn with diminished antimicrobial activity by neutrophils and macrophages, decreased antigen presentation by antigen-presenting cells, particularly dendritic cells, and reduced NK killing, and with a fairly compromised adaptive cellular immune response. In other words, the innate immunity and B cell, Th1, Th2, and Treg activities are all affected, to a variable extent, over the lifetime. Therefore, young and old immune systems are not highly efficient at dealing with infections (e.g., viral) in comparison to those of young adults.

## 3. Immune Response to the Different Types of Vaccines in Children

To date, limited concern has been raised against the commonly used COVID-19 vaccines (Pfizer-BioNTech, Moderna, and COVAVAX) in children, taking into consideration that the immune response in children is stronger than in adults (see below Section 3.2) [44,63,64]. This is reflected during vaccination by more pain and swelling at the site of injection over a few days and possible fever to a mild-to-moderate degree.

All age groups, from neonates of one day of age up to 15-year-old children, are susceptible to COVID-19 infection. The disease seems to be less severe in children than in adults because of the good competency of the immune system in children. It has been reported that 21% of children show obvious clinical manifestations of COVID-19 vs. 69% of adults. Furthermore, it has been found that children aged less than 15 years are less infectious than adults, since they commonly have a lower viral load. Children also have frequent viral respiratory tract infections, including with other coronaviruses, and different types of vaccinations such as BCG can induce trained innate immunity (see below, Section 4.1) [5,68]. BCG provides cross protection against other pathogens (other than *Mycobacterium tuberculosis*) such as SARS-CoV-2 infection [68]. BCG has immunomodulatory properties which can induce the cellular immune response as well as activation of components of the innate immune system, such as monocytes, macrophages, and NK cells [5,68]. 

### 3.1. Immune Response to COVID-19 Vaccines 

COVID-19 vaccines trigger immunological responses. mRNA-based adenoviral vectors or inactivated virus can produce a coordinated innate and adaptive response, specifically reducing the damage caused by a real SARS-CoV-2 infection [69,70].

The innate immune response is the first line of defense against SARS-CoV-2. APCs, including monocytes, macrophages, and dendritic cells (DCs), may recognize PAMPs generated by SARS-CoV-2 via their pattern recognition receptors (PRRs), including TLR3 and -7. This increases the activity of intracellular signaling pathways, leading to the manufacture of type I and III interferons (IFNs) which, in turn, encourage innate immune cells to produce proinflammatory cytokines and chemokines. This causes the recruitment and activation of neutrophils, more APCs, and other innate immune cells, such as natural killer (NK) cells [71]. In all cases, identification of the spike protein stimulates the innate immune system and interferon pathways, demonstrating antiviral properties and resulting in a rise in cytolytic T cells and antibodies against SARS-CoV-2 [72].

During anti-COVID-19 vaccination, the adaptive immune response is triggered following viral uptake and antigen processing by the APCs. These cells transmit the viral antigen to B cells, which then differentiate into plasma cells that make antibodies. The neutralizing antibodies (nAbs) attach to several viral proteins, including as the spike (S) protein and neutralize their effectiveness. Other antibody-mediated antiviral effects include antibody-dependent cellular cytotoxicity, antibody-dependent cellular phagocytosis, and antibody-dependent complement activation [71,72,73].

Cytotoxic CD8+ T lymphocytes (killer cells) destroy virally infected cells by generating granzymes and perforin and by expressing the Fas ligand (FasL). All of these methods enhance the removal of virus particles by cellular death. Populations of CD4+ T cells are also involved in the SARS-CoV-2 immune response. Follicular helper T cells (TFH) and Th2 CD4+ T cells both assist B cells in the generation of specific antibodies. Moreover, Th1 and Th17 CD4+ T cells contribute to the inflammatory response and viral elimination. In SARS-CoV-2 infection or vaccination, CD4+ regulatory T cells regulate the immune system through the production of anti-inflammatory cytokines and contact-mediated cellular suppression [71].

**Figure 2:**
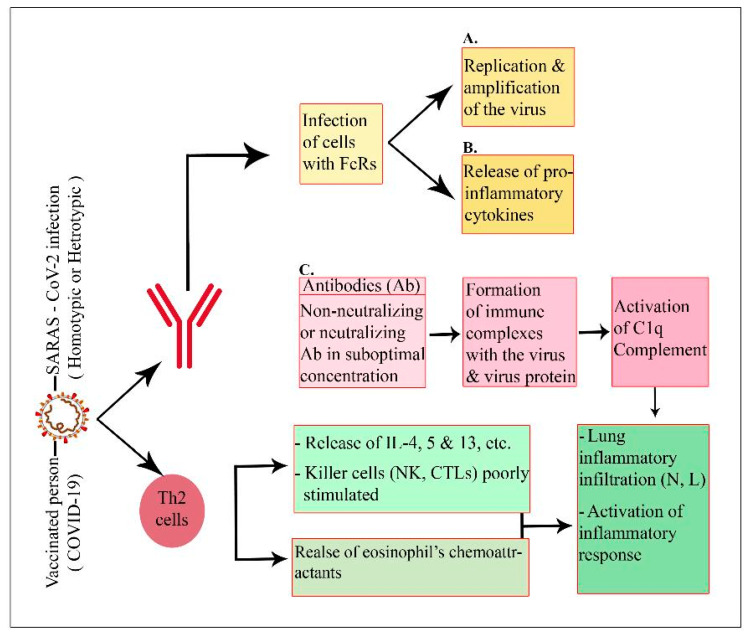
Immune response during SARS-CoV-2 infection of a vaccinated person. FcRs, Fc receptors; IL, interleukin; NKs, natural killer cells; CTLS, cytotoxic T lymphocytes; N, neutrophils; L, lymphocytes.

When the homologous virus enters an inoculated body under physiological conditions, it is neutralized or eliminated by vaccine-induced neutralizing antibodies (Abs) and/or particular T cells. Vaccines predominantly induce non-neutralizing Abs or low titers of neutralizing Abs and/or type 2 T helper cell (Th2 cell) responses in the context of vaccine-associated illness enhancement. When these immunized individuals are exposed to homotypic or heterotypic serotype viruses, the antibodies instantly recognize the pathogens and cause antibody-dependent illness (Figure 2). The virus–antibody complexes that bind to the Fc receptor (FcR) on cells, such as dendritic cells and monocytes, subsequently entering internalization via an ADE, can activate three pathways. Upon initial infection, the virus may induce a malignant immunological response, resulting in the release of proinflammatory cytokines (IL-1ß, IL-6, IL-8, IL-17, and TNF-α), (Figure 1 and Figure 2) [12,69]. Second, the creation of complexes can activate the classical complement, leading to an increase in inflammatory reactions. Third, this vaccine-associated illness enhancement may also involve a Th2-biased immune response that results in the formation of certain antibody isotypes. Additionally, Th2 cells secrete the eosinophil chemoattractant, causing eosinophil infiltration and proinflammatory cytokine synthesis in the lungs. All three of these pathways contribute to the development of acute lung damage or acute respiratory distress syndrome. It is worth mentioning that natural killer (NK) cells and CD8^+^ cytotoxic T lymphocytes are poorly stimulated in Th2-cell-skewed immune responses [74].

### 3.2. Safety of Anti-COVID-19 Vaccines from Infancy to Adolescence

In order to understand the efficacy and safety of the different anti-COVID-19 vaccines, their effects and the protective immunity produced are categorized according to the type of vaccine [69,70,71,72].

#### 3.2.1. Live-Attenuated Vaccines

Live-attenuated vaccines are prepared from whole SARS-CoV-2 virus and have the ability to stimulate the immune system by inducing TLRs. The generation of weakened vaccine versions can stimulate innate immunity TLRs, such as TLR3, TLR7/8, and TLR9. This activation can bridge the gap between the latter type of immunity to the adaptive immunity, which involves B cells, CD4 helper T cells, and CD8 cytotoxic T cells [11,69]. Intranasal anti-COVID-19 vaccines may represent an option for this purpose. Mucosal-generated immunity can protect the upper respiratory tract against SARS-CoV-2 infection. The Codagenix Company with the Serum Institute of India produced a modified SARS-CoV-2 virus (live attenuated) as an intranasal vaccine (COVI-VAC) which is under clinical trial (Table 2) [69,70].

#### 3.2.2. Inactivated Vaccines

Inactivated vaccines are simple to manufacture from whole SARS-CoV-2 virus, express conformation-dependent antigenic epitopes, and can be combined with adjuvants to boost their immunogenicity. These vaccines produce a less potent immune response than the other type of vaccine, and booster/additional doses are required, because they do not produce long-lasting immunity; thus, reinfection is possible. Since the entire virus is presented to the immune system, it is possible that immune responses will target not only the spike protein, but also other viral epitopes. The effectiveness and safety of these vaccines have been proven in the laboratory [50,69]. The most common inactivated vaccines in clinical use are Sinovac (Coronavac), Sinopharm (BIBP and WIBP), Covaxin, and Valneva SE (Table 2) [69,70,71,72,73,74]. 

#### 3.2.3. DNA Vaccines

DNA vaccines enhance humoral and cellular immune responses and are stable and easily prepared in large quantities. A recently applied DNA vaccine is ZyCoV, which was shown to yield a vaccine efficacy of 66.6% [75].

#### 3.2.4. RNA Vaccines

Both Pfizer/BioNTech and Moderna’s vaccines employ mRNA technology to transmit the genetic code of the SARS-CoV-2 virus to the body without altering the cells of the body. This is in contrast to DNA-based vaccinations, which may result in host genomic anomalies. mRNA-based vaccines are an efficient and safe way to induce robust immune responses that can resemble a real SARS-CoV-2 infection. Near to the injection site, antigen-presenting cells (macrophages and dendritic cells) take up an administered mRNA vaccine. Inside these cells, the mRNA utilizes the host cell’s ribosomes to make the SARS-CoV-2 spike protein, which is subsequently expressed on the cell’s surface, inducing humoral and cellular immune responses [76,77].

#### 3.2.5. Subunit Vaccines

Subunit vaccines do not contains live components of viral particles. They are safe with fewer side effects than other vaccines. Viral proteins or protein fragments of the SARS-CoV-2 virus, particularly the S protein, are used in the preparation of these vaccines [69,78]. These vaccines have weak immunogenicity, are safe, and have few side effects. Adjuvants (aluminum sulfate “alum” alone or alum with CpG) are mixed with them in order to potentiate the immune response against them [69].

#### 3.2.6. Vector Vaccines 

Vector vaccines can infect antigen-presenting cells (APCs) directly. These vaccines are physically and genetically stable. These vaccines are considered to be live-attenuated vaccines that can integrate into the host genome, and therefore, concerns have been raised regarding possible cancer development [77]. They produce a robust immune response against the SARS-CoV-2 virus and are not recommended for use in infants or children, and they are also not used in pregnant or immune-deficient individuals.

There are nine types of viral vectors that have been used for the preparation of different vaccines, including five non-replicating vectors and four replicating ones. The former group, which are used in the preparation of COVID-19 vaccines, includes AZD 12232 (Oxford/AstraZeneca), GNJ-7836735 (Johnson & Johnson), Ad5-nCoV (CanSino Biologics of China), and Ad5 and Ad26 (Sputnik and Sputnik Light vaccines of Gamaleya Research Institute of Russia) [69,70]. The latter group includes different adenovirus vectors (Adv), the modified vaccinia virus Ankara (MVA), the parainfluenza virus vector (PIV), and the lentivirus (LV). These replicating vectors are engineered by the deletion of their replicating ability by gene removal. However, none of these vectors have been applied for the preparation of COVID-19 vaccines, since they induce a robust and persistent immune response and the generation of muted viral particles [78]. Their efficacy is derived from their capacity to infect cells, which enables them to provoke significant immunological responses. However, there may be pre-existing immunity to the vector, and only a small number of CoV antigens may be exposed to the host immune system. Integration into the host genome may cause cancer, and redeveloped immunity to the vector is a disadvantage [69].

### 3.3. Different Immunological Mechanisms after COVID-19 Vaccination

Table 2 summarizes the different mechanisms of the immune response against the different types of common globally distributed vaccines. Four mechanisms are shown: against mRNA, vectored vaccines, inactivated vaccines, protein subunit vaccines and live attenuated vaccines. Basically, all types of vaccines generate protective mechanisms against SARS-CoV-2, but these are of variable magnitude and effectiveness [72,73,79].

**Table 2:** Immune response against the commonly used vaccines and their types and manufacturers modified from [69,78].

Vaccines	Manufacturers	Immune Response
**mRNA** - BNT162b2 - mRNA-1273	- Pfizer/BioNTech + Fosun Pharma - Moderna + National Institute of Allergic and Infectious diseases	Expression of the viral S protein encoded in the mRNA and antigen presentation; induction of IFN I release, stimulating Th1 response, antibodies, and memory T and B cell generation
**Adenoviral Vectored** - AZD122 (ChAdOx1-S) - Ad26.COV2 S - Sputnik V (rAd26-S + rAd5-S)	- AstraZeneca + University of Oxford - Janssen Pharmaceutical by Johnson & Johnson - Gamaleya Research Institute of Epidemiology and Microbiology	Emulation of viral infection; induces expression of IFN, antibodies, and memory T and B cells, along with T CD8 activity
**Inactivated** - CoronaVac - Sinopharm - Covaxin - Valneva	Chinese Sinovac Biotech. BIBP B Beijing BIBP and WIBP Indian Baharat Biotech French Biotechnology Com	Similar to adenoviral vectors; induction of IFN, memory T and B cells, antibody production, and T CD8 activity
**Protein Subunit** - PREVENT-19 (NVX CoV-2373)	Novavax	Enhanced humoral responses and B and T memory cells
**Live-Attenuated** - COVI-VAC	- Codagenix and Serum Institute of India	Induces antibody-based humoral and T lymphocyte-based cellular immune responses

### 3.4. Vaccination of Children Aged 5–15 Years 

The FDA in the USA has granted emergency approval (EUAs) for three vaccines to be used in children: two mRNA vaccines (Pfizer/BioNTech and Moderna) and one vectored, the Ad26 vaccine (Johnson & Johnson). On 10 May 2021, the FDA approved the Pfizer/BioNTech vaccine for use in adolescents aged 12–15 years, and on 29 October 2021, it was approved for children aged 5–11 years [2]. For children aged 5–11 years, the Pfizer/BioNTech (BNT 162b2) and Moderna (mRNA 1273) vaccines are given in two doses, three weeks apart, in lower doses (10 µg) than that used for older children (30 µg) [3,80,81]. This protocol of vaccination provides protection of approximately 91% for these children [3,81]. Vaccination in this age group might not produce a strong immune response and, therefore, the CDC recommends an additional dose be given 28 days after the second dose of the vaccine [3,81].

For children aged 12–15 years, again, the Pfizer/BioNTech vaccine is given in two doses, three weeks apart (or six weeks apart), but in doses similar to that given to adults (>16 years). This vaccination is 100% protective in this group of children. In India, the Covaxin adjuvanted inactivated vaccine and ZyCoV (novel DNA) vaccine have also been approved for use in children aged 12–17 years [3,75,80,81].

Moreover, Chinese authorities have approved the use of two inactivated vaccines (Sinovac-CoronaVac and BBIBp-CorV) for children as young as three years of age [81]. Inactivated vaccines are more suitable for very young children, since their immune systems are still in the process of evolution toward maturity (see above). Several trials on COVID-19 vaccines are underway for infants as young as six months of age, but validated results have not yet been published [81].

Among all subtypes of vaccines, mRNA-based vaccines are the most successful, because they are safer, can elicit a greater immune response, and can mimic natural infection [69,70,71,72,73,76,79]. mRNA vaccines have the disadvantage of needing particular storage and shipping conditions. Due to the fact that inactive vaccinations contain the full virus cell, their side effects are severe, and a booster dosage is required to maintain immunity (Table 2) [4,69,71].

### 3.5. Vaccination for Children Aged Less Than Five Years

In youngsters, the immune system is undergoing a developmental process while vaccination provides protection, and both can occur simultaneously [4]. The US Food and Drug Administration approved the emergency use of the Moderna and the Pfizer/BioNTech COVID-19 vaccines for the prevention of COVID-19 in infants as young as six months of age on 17 June 2022 [82]. In addition, on 3 August 2022, the Therapeutic Goods Administration of Australia (TGA) began to evaluate a request from Pfizer Australia Pty Ltd. to increase the use of its COVID-19 vaccine (COMIRNATY) to a three-dose course for children aged six months to less than five years [83]. A lower vaccine dose (3 µg in 0.2 mL) will be evaluated for younger children. This is in comparison to a dose of 10 µg for 5–11-year-olds and 30 µg for those aged 12 years and older (see above, Section 3.4). In addition, on 19 July 2022, the TGA granted provisional approval for the use of a pediatric dose of the Moderna COVID-19 vaccine (SPIKEVAX) in children aged six months to less than six years.

Children under five years of age are much less vulnerable to the coronavirus than adults, but they are not invulnerable to its serious consequences. In the United States, more than 460 children aged under five years have died from COVID-19 [2]. These children have died at a higher rate than children aged 5–11 years. Therefore, it is difficult to make decisions concerning the administration of COVID-19 vaccines to this group of children. It is frustrating to report on children who have died from COVID-19 because they were unvaccinated or had complications from vaccination [2,81,84].

The detection of new variants with increased transmissibility and reduced sensitivity to vaccines and a decreased capability to prevent infection has led to focus on vaccinated groups where the immune response may be compromised [85].

Vaccination of these children could reduce the spread of the disease and help to protect other vulnerable individuals. In light of the emergence of the delta and omicron variants, herd immunity protection seems to be of limited value. It looks like the virus will stay with humanity, and the best practical method of protection is vaccination [85]. Currently, the vaccine can be recommended for children aged under five years, both because it is authorized for use and because the available data show acceptable safety and efficacy levels. It is reassuring that severe outcomes are rare in children, but they do occur. However, the available global data show that there are risks associated with vaccination in children aged 5–18 years occur but on a very limited scale. Similarly, the data on children aged under five years follow the same pattern, so it is recommended that all children are vaccinated, unless there are contraindications [85,86].

### 3.6. Virology of SARS-CoV-2 and the Generated Immunity against Viral Components

The 3′ end of the viral genome encodes the envelope (E), membrane (M), spike (S), and nucleocapsid (N) structural proteins of SARS-CoV-2 [87]. The S protein promotes the binding of SARS-CoV-2 to ACE2 on the host cell, facilitating viral entrance and pathogenesis. Considered a key virulence factor, the E protein plays a role in the secretion of inflammatory factors. The N protein is responsible for mRNA transcription and RNA replication and produces the nucleocapsid. The M protein plays an important role in viral assembly. Due to its vast genome, the virus is less dependent on the host during replication and can reproduce independently of the host genome. In addition, the RNA-dependent RNA polymerase (RdRp) gene allows the virus to reproduce its genome in the cytoplasm of the host. SARS-CoV-2 enters the cell by endocytosis after attaching to its receptor, releases viral RNA into the cytosol, and exploits the cell’s replication machinery. In the last phase, the endoplasmic reticulum and Golgi body combine the viral proteins and RNA genome into virions, which are then expelled from the cell via exosomes [69,87].

#### 3.6.1. Role of ACE Receptor Expression during COVID-19 Infection and Vaccination

Approximately 80% of all human ACE2-expressing cells are type II alveolar cells, with the remainder being distributed among the nasal mucosa, upper respiratory tract, endothelium, heart, kidney, and gut cells [13,14,88,89,90]. To be successful against nonrespiratory systemic viral infections, the vaccine must protect the recipient from the virus’s systemic dissemination by inducing IgG production. The expression of the SARS-CoV-2 entry receptor ACE2 increases from childhood through adulthood and then decreases with age [91]. It is present in most tissues; however, its expression is highest in type 2 pneumocytes [92]. It has a protective function, and the exact relationship between ACE2 and the severity of COVID-19 disease is unknown. Adults with comorbidities, such as hypertension and diabetes, have decreased ACE2 activity [92]. Studies on animals have demonstrated that ACE2 protects against lung damage caused by SARS-CoV-2 [91]. Affinity for ACE2 may influence the severity of an illness. It is hypothesized that, in children, ACE2 has a decreased affinity for SARS-CoV-2, hence inhibiting viral entrance into host cells [91].

#### 3.6.2. Classes of Antibodies Produced in Response to COVID-19 Vaccines

The variation in individual immune responses to COVID-19 infection demonstrates that the use of a single immunological strategy when designing a vaccine is not adequate for achieving long-lasting protection for all people [93,94]. Vaccines must swiftly and reliably reproduce specific immune responses that induce viral clearance.

The lower respiratory tract is mostly protected by IgG, whereas the upper respiratory tract is primarily protected by secretory IgA. Natural infection with respiratory viruses causes both a systemic immune response and a mucosal immunological response, which are, respectively, mediated by IgG and IgA [95,96]. Vaccination administered intramuscularly or intradermally induces a substantial increase in blood IgG but not mucosal IgA. Vaccination administered intranasally can effectively induce mucosal antibody responses, hence conferring sterilizing immunity to the upper respiratory tract. However, systemic immune responses to this sort of vaccination are frequently weak. Currently, the majority of SARS-CoV-2 vaccine candidates under clinical research are delivered intramuscularly and focus on the response to IgM, IgG, or total immunoglobulin in the blood [95,96].

#### 3.6.3. Viral Mutations and Childhood Immunization

Multiple new COVID-19 strains have emerged in various regions of the world since December 2020 [97,98,99]. The new strains are more contagious and virulent, and they can cause increased morbidity and mortality. At present, there is no evidence that youngsters are susceptible to the new variations. There is serious concern that changes in the spike glycoprotein may render the vaccine-induced antibodies ineffective or subneutralizing [100]. Current COVID-19 vaccines are still thought to be effective against the N501Y variant, but their efficacy may be diminished against other novel variants of concern [99,100]. The Pfizer/BioNTech COVID-19 vaccine was shown to decrease the probability of infection with the omicron version of SARS-CoV-2 by 31% in children aged 5–11 years and by 59% in adolescents aged 12–15 years [101]. In addition, it has been reported that the vaccine protects adolescents against infection with the delta variant by 87%. These variants showed a proliferative pattern before youngsters had access to the vaccines [99,100,101].

In contrast, sera from participants aged over 80 years have been shown to have a lower neutralization efficacy against the B.1.1.7 (Alpha), B.1.351 (Beta), and P.1. (Gamma) variants of concern (VOC) than against the wild-type virus and are more likely to lack neutralization against VOC following the initial dose [102]. Regardless of age, VOC neutralization is apparent following the second dose. After the first treatment, older responders (whose blood displays neutralizing action) have a higher concentration of SARS-CoV-2 spike-specific memory B cells than nonresponders. Interferon- and interleukin-2 production by SARS-CoV-2 spike-specific T cells is diminished in older individuals, and both cytokines are mostly produced by CD4 T cells. Consequently, the elderly are a high-risk population, and extra methods to improve vaccination responses in this population are necessary [102,103], particularly if VOC are widespread.

### 3.7. Safety of COVID-19 Vaccines for Infants and Children 

Concerns have been raised about the use of COVID-19 vaccines in children, considering that the immune response in children is stronger than in adults [101,102,103,104]. The direct health benefits of vaccinating children and adolescents are less than in adults, because there have been fewer severe cases and deaths in the former group than in the latter group. However, the risk of myocarditis development is worth mentioning. Although rare, this can occur after the second dose of the Pfizer/BioNTech vaccines (BNT162b2 or mRNA-1273) in adolescents aged 12–17 years [105,106,107,108]. The FDA’s model also predicts that the benefits of the vaccine will outweigh its risks in children aged 5–11 years [80,107].

#### 3.7.1. Parental Hesitancy towards Vaccination of Their Children

Children’s immunization against COVID-19 is a topic of public concern and controversy, and in countries where COVID-19 vaccines are available, various vaccination strategies are in use. Compared to COVID-19 immunization in adults, the BNT162b2 mRNA vaccine has been demonstrated to have superior effectiveness in 5–15-year-old children and adolescents [109]. Recent studies on the immunogenicity and safety of mRNA vaccines have been undertaken in these age ranges. Studies have also demonstrated the immunogenicity and safety of the inactivated CoronaVac vaccine in children aged 3–11 years [110]. Studies on nonhuman infant primates using mRNA vaccinations revealed robust and lasting antibody and T cell responses [111]. mRNA and inactivated COVID-19 vaccines have been licensed for emergency use in young children and older adolescents by regulatory bodies in numerous countries and rolled out in others on the basis of these studies.

The decision to propose and administer COVID-19 vaccines to adolescents and younger children is typically based on benefit–risk evaluations [112,113,114,115]. In weighing the necessity of vaccination in children, it is essential to keep in mind that the majority of children remain asymptomatic, 6% are hospitalized with 13% of those hospitalized meeting the criteria for severe disease with a fatality rate of 1%, and others suffer from prolonged symptoms (long COVID) and could benefit from vaccination [116]. Overall, the benefits are greater for vulnerable children who are at risk of developing severe COVID-19 as well as for the protection of the entire community. Although children are not a high-risk population for SARS-CoV-2 transmission, vaccination would minimize the risk of infection and sickness for their contacts. If those contacts are susceptible and respond poorly to immunization due to immunosuppression, this would be quite advantageous. It has been proposed that children should be required to get the COVID-19 vaccine in order to achieve a high vaccination coverage and herd immunity [117,118,119]. However, given that parenteral hesitation toward children’s vaccination continues to be high in many nations, the chance of achieving herd immunity, particularly for strains of concern, is becoming more remote, and the case for mandating the vaccination of children has become weaker in recent years.

A further advantage of COVID-19 immunization for youngsters is that it would permit control mechanisms to be relaxed, leading to an increase in fearless social interactions. [113]. Therefore, a number of advantages of COVID-19 immunization for healthy youngsters can be recognized, albeit they are less evident than those for adults. Despite the fact that clinical trials have demonstrated the safety of mRNA and inactivated COVID-19 vaccines, more safety data are required. Immunization of children against COVID-19 is likely to be promoted more in the foreseeable future, given the rising recognition of the benefits and safety of vaccination among both adults and children.

#### 3.7.2. Mechanisms of the Adverse Effects of Vaccination in Adults and Children

To clarify the issue of the adverse effects of vaccination, as can occur in adults, there are several mechanisms that can be discussed. These possibilities, although rare and limited, are worth mentioning and are also under consideration in current and future evaluations. These effects have a variable range and can occur from the very first day of vaccination to weeks or even months later. These adverse effects, which are mainly attributed to immunological reactions, are seen both in adults and children [120].

In adults, these include systemic inflammatory response syndrome (SIRS) proportional to the cytokine storm; wide interaction between S viral proteins and widely expressed ACE2 receptors; interaction between S viral proteins and platelets and/or endothelial cells, leading to clotting, bleeding, and neurological effects; mast cell activation syndrome (MCAS); and generalized lymphadenopathy. On the contrary, the most important mechanisms for adverse effects in children are commonly classified as hyperinflammation, hypercoagulation, allergic, and neurological. These effects show the relationships with blood disorders, neurodegenerative diseases, and autoimmune diseases [120].

The mechanisms associated with these effects in adults and children can be attributed to the following interactions. The S protein utilized in vaccinations has the potential to be toxic and pathogenic to the body, harm the vascular endothelium, suppress the expression of the ACE receptor, and elevate levels of angiotensin II [121]. This increases coronavirus-induced vascular cell dysfunction [122], elicits functional changes in cardiac vascular pericytes, stimulates ERK1/2 phosphorylation/activation through the CD147 receptor, binds to neural phospholipids and causes their mechanical destabilization and permeabilization, and crosses the blood–brain barrier in mice [123]. Based on the discovery that anti-SARS-CoV-2 protein antibodies cross-react with 28 of the 55 diverse human tissue antigens, it has been suggested that SARS-CoV-2 spike proteins can also induce a proinflammatory response in brain endothelial cells and worsen autoimmunity in individuals who already have autoimmune diseases [124].

Additionally, in both intradermal and intranasal immunization, the lipid nanoparticle (LNP) layer encasing the inoculant’s mRNA is highly inflammatory [125]. Polyethylene glycol (PEG), a vaccine ingredient, is also thought to be a factor in anaphylactic reactions to the Pfizer/BioNTech mRNA COVID-19 vaccine [126]. Due to exposure to common items containing PEG, humans are susceptible to the development of anti-PEG antibodies, which might lead to an increase in allergic reactions after subsequent vaccination [127]. Additionally, investigations on post-inoculation subacute thyroiditis and post-inoculation thyroid hyperactivity have suggested that the LNP shell’s components may lead to the autoimmune/inflammatory syndrome caused by adjuvants (ASIA) syndrome, as shown by studies on post-inoculation thyroid hyperactivity [128] and post-inoculation subacute thyroiditis [129]. It has been demonstrated that ChaAdOx1 (AstraZeneca’s recombinant adenovirus vaccine candidate against SARS-CoV-2) stimulates brain cells to create COVID-19 spike proteins, which may trigger an immune response against brain cells or spike protein-induced thrombosis. This may help to explain the strange occurrences of fatal cerebral venous sinus thrombosis (CVST) linked to COVID-19 vaccines based on viral vectors [130,131].

In conclusion, all of the above-mentioned mechanisms have been derived from research studies, and the collected clinical data do not negate the worldwide usage of anti-SARS-CoV-2 vaccines, since they are rare and of limited concern. The advantages of vaccination are overwhelmingly greater than the limited-scale adverse effects.

## 4. Immune Response to SARS-CoV-2 Infection

### 4.1. COVID-19-Generated Immunity in Neonates to Adolescents

The majority of children with COVID-19 have minimal or even no symptoms, but some may have severe or protracted symptoms (long COVID), in which case vaccination may be helpful [116]. It is important to note that children aged 10–14 years are less susceptible to SARS-CoV-2 infection than adults [3,5,101,102,103,104]. In order to attain a high vaccination coverage into adolescence and to boost herd immunity, COVID-19 vaccination of children has recently been suggested, starting at five months of age [82,83,117,118,119].

The initial line of defense against SARS-CoV-2 primary infection in children is innate immunity [132]. While opposing reactions do happen in mild disease, the interferon (IFN) response is decreased in severe instances with a high inflammatory response [133]. Children with both severe and mild pediatric COVID-19 disease have been shown to have high levels of inflammatory cytokines [134,135,136,137]. This conclusion might be connected to the potential contribution of children’s previously acquired trained innate immunity [138,139]. Similar characteristics involving the activation of different cells, such as monocytes and dendritic cells (DCs), as well as brief decreases in the concentrations of lymphocytes, monocytes, DCs, and NK cells, have been seen after the onset of COVID-19 symptoms in both children and adults [138,140]. When compared to adult COVID-19, neutrophil activation is lower in pediatric COVID-19 cases, which can reduce tissue damage and inflammation [141]. The high percentages of circulating lymphocytes in healthy youngsters, on the contrary, may allow them to better regulate illness [142]. The innate immune system plays a major role in the early regulation of SARS-CoV-2 replication during primary infection [132]. A reduced initial interferon (IFN) response, followed by unchecked and persistent inflammation, is characteristic of severe illness [133]. The key question is whether children have a more robust innate immune response, which can more effectively inhibit viral replication, or whether they mount a less intense inflammatory response that results in fewer and milder symptoms. In general, cytokine levels are lower in children [134], and inflammatory cytokines have been found to be present in lower levels in children with acute respiratory distress syndrome than in adults (Table 3) [143].

Children with COVID-19 were previously said to have clinical indicators of inflammation that were either undetectable or modest [144,145,146,147]. Later studies on hospitalized children and adults, however, found comparable or higher systemic cytokine levels [148]. Additionally, both children with mild disease [138] and those with severe instances of pediatric COVID-19 [135,136,137] have shown significant levels of inflammatory cytokines. Furthermore, it has been shown that children, as opposed to adults, have a more robust nasal innate immune response, including higher levels of IFN-γ and IFN-α. According to this research, children may exhibit stronger early-stage antiviral responses at the mucosal level [148]. Children’s defense against COVID-19-related illnesses may be aided by a strong and effective IFN response [149]. This situation may also be influenced by a potential function for trained innate immunity in the prevention of SARS-CoV-2 infection in children brought on by prior immunizations or frequent infections [139].

Analyses of peripheral blood immune cells in the early stages of symptoms in children and adults have revealed similar characteristics, including the activation of monocytes and dendritic cells (DCs) and momentarily lower numbers of lymphocytes, monocytes, DCs, and NK cells [138,140]. Neutrophils appear to be less active in juvenile COVID-19 instances than in adult cases, which may reduce tissue damage and inflammation [48]. Additionally, children have more circulating lymphocytes than adults do, which may help with illness management [142]. In children, these cells may be recruited at the site of infection earlier and in greater numbers than in adults. (Table 4).

**Figure 3:**
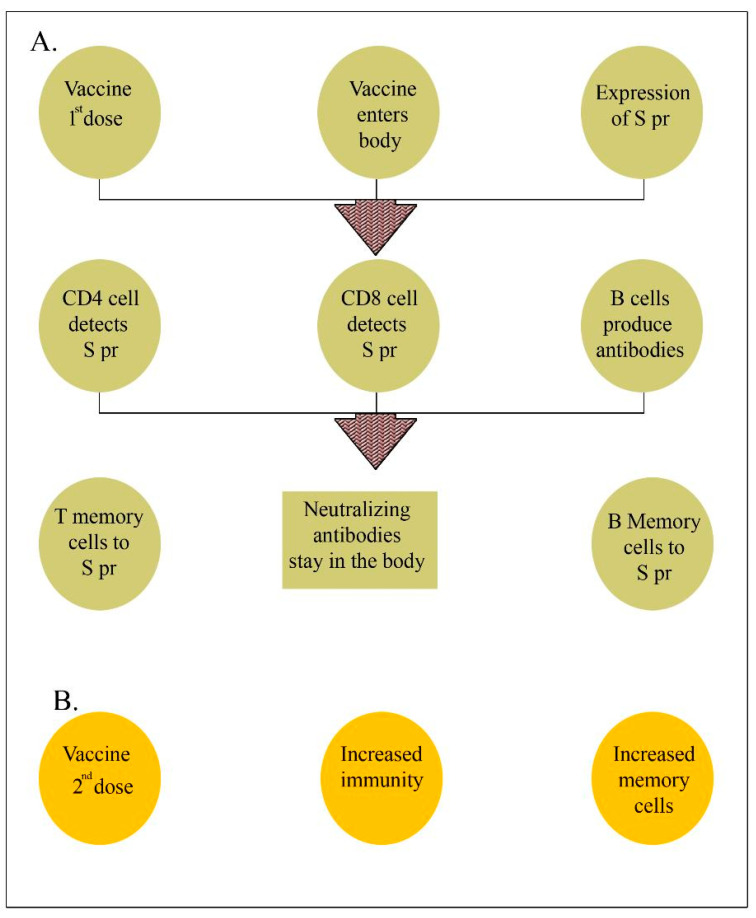
Immune system status after the first and second doses of the anti-COVID-19 vaccine. SPr, S protein. (**A**): shows the stimulation of T cells (CD4 and CD8) and B cells and the generation of neutralizing antibodies and memory cells. (**B**): shows enhanced immunity and memory cell activity after the second (booster) vaccine dose.

It is now well recognized that children can mount a robust neutralizing antibody response to SARS-CoV-2 [150,151,152,153]. Initial reports from small pediatric cohort studies have shown lower serum neutralizing activity compared to adults [148,154], as well as a reduced antibody response to the spike protein [154]. Additionally, it has been noted that, regardless of the severity of the illness, children have lower levels of SARS-CoV-2 antibodies, with a substantially higher percentage of these antibodies targeting nonstructural viral proteins [155]. In children and adults with mild COVID-19, further investigations have revealed a comparable functional antibody profile, including phagocyte and complement-activating IgG [151,156]. Additionally, compared with adults, children show a quicker beginning of the antibody response to the receptor-binding domain (RBD) and a quicker emergence of the peripheral blood B cell transcriptomic signature. Children’s faster B cell responses to SARS-CoV-2 could possibly help them to better contain the virus and lessen symptoms. Children’s levels of neutralizing antibodies and antibody-secreting B cells exhibit an inverse relationship with the viral load at seven days after the onset of symptoms (Figure 3) [157].

Additionally, greater prolonged antibody responses and levels of somatic mutations in memory B cells are linked to a quicker recovery from symptomatic COVID-19 in adults [158]. Children have lower levels of anti-SARS-CoV-2 IgG six months after infection than their infected parents at the same time [159]. Following a moderate or asymptomatic infection, several trials on children and adolescents have revealed robust and long-lasting antibody responses to SARS-CoV-2 [160]. In terms of cell-mediated immunity, only a small number of papers have described decreased or comparable levels of SARS-CoV-2-specific T cells in children (Table 5) [36,63,71,148,161].

In conclusion, the degree and severity of COVID-19 infection in an individual may depend on the development of an antiviral type I IFN response early after infection. In individuals with severe COVID-19 infection, it has also been discovered that the number of autoantibodies against IFN-I increases proportionally with age. Therefore, it is advised that children with APS-1 who have been exposed to SARS-CoV-2 should be treated by removing neutralizing anti-IFN-I autoantibodies by plasma exchange. Severe COVID-19 problems result from early IFN-I and/or IFN-III responses that fail to limit SARS-CoV-2 replication. The occurrence of severe COVID-19 pneumonia in children and young people is caused by genetic alterations in the type I IFN gene. Furthermore, the differences between children and adults’ tissue immune cell composition and baseline antiviral gene expression in epithelial cells demonstrate that children’s antiviral responses are far more potent.

**Table 4:** Key components of the adaptive immune response against SARS-CoV-2 infection.

Immunocompetent Cells	Immunological Elements	Functions
B cells	SARS-CoV-2 neutralizing antibodies	- Viral neutralization
		- Antibody-dependent cellular cytotoxicity (ADCC)
		- Antibody-dependent cellular phagocytosis (ADCP)
		- Complement activation (ADCA) [71,150,151,152,153,154,155,156]
T_FH_ cells	CD4-positive cells	- Interact with B cells to enhance survival and provide cellular help for antibody production [11,36,71,148,161] *
CD4 Th1 T cells	IL-2, IFN-γ, and TNF-α	- CD8 T cell help, inflammation, and killing of virally infected cells
CD4 Th2 T cells	IL-4, IL-5, and IL-13	- B cell help and induction of antibody class-switching
CD4 Th17 cells	IL-17A, IL-17F, IL-21, and IL-22	- Inflammation via neutrophil recruitment and activation and innate cell activation
CD4 Regulatory T cell (Treg)	IL-10 and TGF-β	- Suppression of inflammation and other T cells via cytokines or contact-dependent mechanisms
CD8 cytotoxic T cells	Granzymes, perforin, IFN-γ, TNF-α, and FASL expression	- Killing of virally infected cells [29,44,45,46,47,48,63,71,74]

* References are for all CD4 cell subtypes.

### 4.2. Clinical Information Regarding COVID-19 in Children

According to previous research, adults, particularly the elderly, are more likely to contract SARS-CoV-2. Anyone, even infants, can contract this illness [162,163]. Children with COVID-19 are 6.7 years old on average (range: 1 day to 15 years), according to reports [164]. SARS-CoV-2 infection is less common in children aged between 10 and 14 years [12]. Children seem to experience COVID-19 symptoms more subtly than adults. Even though 69% of infected adults have clinical indications, only approximately 21% of babies have obvious symptoms [165].

The most common signs of an upper respiratory infection in children are fever and cough. Fever can range from low to severe. Respiratory discomfort, a sore throat, a runny or clogged nose, weakness, myalgia, and a headache are other clinical signs and symptoms. Furthermore, gastrointestinal issues, such as diarrhea, vomiting, and nausea, have been reported [166,167,168]. Due to their lower viral loads, children are also less contagious; nevertheless, once they reach the age of 15 years, their contagiousness approaches that of adults.

Children are less susceptible to COVID-19 due to the frequency at which they contract viral respiratory tract infections and their possible recent exposure to one of the other major corona virus strains. Angiotensin-converting enzyme (ACE2) protection in children with high levels of COVID-19 may result in less severe sickness [91,92]. Severe COVID-19 may appear in children with underlying chronic conditions, such as immunological deficits, hematological or oncological malignancies, and asthma, because of the persistence of systemic inflammation [77,169].

The characteristic of severe COVID-19 is hyper-responsiveness of the immune system. During the initial immune response to viral infections, type I interferon (IFN) is generated. IFN promotes intracellular RNA breakdown and virus elimination, promotes tissue repair, and initiates a prolonged adaptive immune response. The delay in type I IFN release is assumed to be the root cause of the deterioration in viral control and the episode of hyperinflammation in severe COVID-19 cases [170,171,172]. Furthermore, it can been seen that people with serious illnesses create cytokines erratically and in an uncontrolled manner, which aggravates their symptoms and condition. The cytokine-storm-induced inflammation that compromises the pulmonary vascular and alveolar barrier results in alveolar interstitial thickening, vascular leakage, pulmonary fibrosis, and death [170,171,172].

### 4.3. Severe COVID-19 Immunopathology in Kids and Adults

#### 4.3.1. Innate Immunity’s Function

In adults with severe COVID-19, the innate immune system is hyperactive but ineffective, and the adaptive immune system is suppressed [173]. This can lead to the failure of many organs, severe immune-mediated lung injury, and unchecked production of proinflammatory cytokines (Figure 1 and Figure 2) [173]. High concentrations of circulating neutrophils and monocytes are present in severe adult COVID-19 cases. Inflammatory cytokines are released by classic (CD14+CD16+) and intermediate (CD14+CD16+) monocytes in severe COVID-19 cases [173]. These activated innate cells are extremely uncommon in patients with mild illness or in asymptomatic patients. Nonclassical monocytes (CD14-CD16+), which have anti-inflammatory properties and play a role in maintaining endothelium integrity, are less prevalent in severe adult COVID-19 cases [173,174].

Neutrophil extracellular traps (NETS), which are created by active neutrophils, “trap” infected cells and bacteria. These NETS can also fuel the clotting cascade. Thus, the microangiopathy and thrombosis seen in COVID-19 infection have been linked to NETS [174]. Children with PIMS-TS undergo endothelial cell death and microthrombosis, especially in skin lesions [175]. The underlying microvascular and thrombotic etiology of pediatric inflammatory multisystem syndrome, which is temporally associated with SARS-CoV-2 and severe adult COVID-19, may be comparable.

In COVID-19, the concentration of NK cells is continuously reducing, but this is more obvious in individuals with severe disease [173]. According to one study [176], COVID-19 patients in intensive care units (ICUs) had fewer perforin-producing NK cells than non-ICU patients. In severe sickness, NK cells are not only less functional but also fewer in number. However, the NK cell count is preserved in pediatric COVID-19 cases. Similar decreases in NK cells and nonclassical monocytes have been found in children with PIMS-TS [177].

After typical childhood infections and/or vaccinations, innate immune cells are functionally reprogrammed, allowing for the development of memory cells resembling the adaptive immune system, and this leads to trained immunity in children [178,179,180]. APCs, like DCs, are suppressed by SARS-CoV-2, which indirectly prevents T cells from activating and functioning [174]. DCs produce interferon (IFN) I and III, which are powerful antiviral cytokines that are important in the initial phases of viral infections. IFN suppression, the host’s most effective antiviral defensive mechanism, enables SARS-CoV-2 to bypass the immune system [177].

#### 4.3.2. The Role of Adaptive Immunity

##### The Cellular Components

Lymphopenia and a high neutrophil-to-lymphocyte ratio are observed in severe cases of adult COVID-19 but are uncommon in children [181,182]. Adults have decreased levels of CD4+ and CD8+ naïve and memory T lymphocytes [183]. However, while these are intact in pediatric COVID-19, lymphocyte levels in PIMS-TS are reduced [177,180].

Adult COVID-19 patients have an abnormally high number of CD8+ T lymphocytes [183]. Additionally, those with intermediate illness display a higher SARS-CoV-2-specific T cell response compared to those with severe illness. This shows how intact adaptive immunity is linked to better disease control and recovery [174,183].

T cells from individuals with severe COVID-19 have higher levels of the expression of cell fatigue and inhibitory indicators of programmed cell death, such as PD-1, LAG-3, T cell immunoglobulin, and mucin-domain containing-3 (TIM3) [174,182]. T cell exhaustion is linked to lower effector performance, which implies that humoral response impacts both the functional and quantitative facets of cellular immunity [174,183,183].

##### The Humoral Response

Adult COVID-19 patients develop neutralizing antibodies against the spike glycoprotein (S), which are present in the majority of convalescent individuals [174]. These patients have higher plasma cell counts but lower overall B cell counts [173]. IgG and IgA levels are briefly raised in adults who are asymptomatic or only mildly sick [173]. People with severe COVID-19 have persistently high IgG and IgA levels. Stronger concentrations of IgG and IgA, particularly in neutrophils that express surface Fc receptors, can also maintain innate cell activity, which may be correlated with stronger neutralizing antibodies [184,185]. An early rise in IgM levels was noticed in asymptomatic, mild, and severe patients [173]. Pediatric COVID-19 cases have been shown to have early-stage IgM elevations, persistent IgG levels, and falling IgA levels [173,174].

On the other hand, the IgG and IgA SARS-CoV-2 immune complexes also activate the complement system through the MBL and the classical pathways as part of humoral immunity. MAC complexes (C5b-9) lyse cells after complement activation, promoting phagocytosis and the formation of NETS [174].

Conversely, children with PIM-TS lack IgM [184]. Further evidence that PIMS-TS is a clinical condition that manifests weeks after an acute infection has been found in these children and is provided by the SARS-CoV-2 PCR results, which were negative [184]. Children with PIMS-TS are more prone to monocyte activation and long-lasting hyperinflammatory activity due to their high IgG levels and long-lasting Fc receptor-binding capacity [185].

#### 4.3.3. Anamnestic Responses to SARS-CoV-2

The progression of severe adult COVID-19 is rather gradual (i.e., 19 days after the onset of symptoms in fatal cases) [186,187], showing that the protective role of memory B cells and T cells requires days to develop [187]. Virus-specific memory CD4+ and CD8+ T cell responses remain active for a minimum of six months, but virus-specific antibody responses drop after three months of infection [188]. Furthermore, memory B cell responses to SARS-CoV-2 have been shown to form at between 1.3 and 6.2 months after infection, corresponding to the persistence of viral antigens in enterocytes [189]. Immunosenescence may also predispose individuals to ineffective viral clearance, hence accelerating the course of COVID-19.

**Figure 4:**
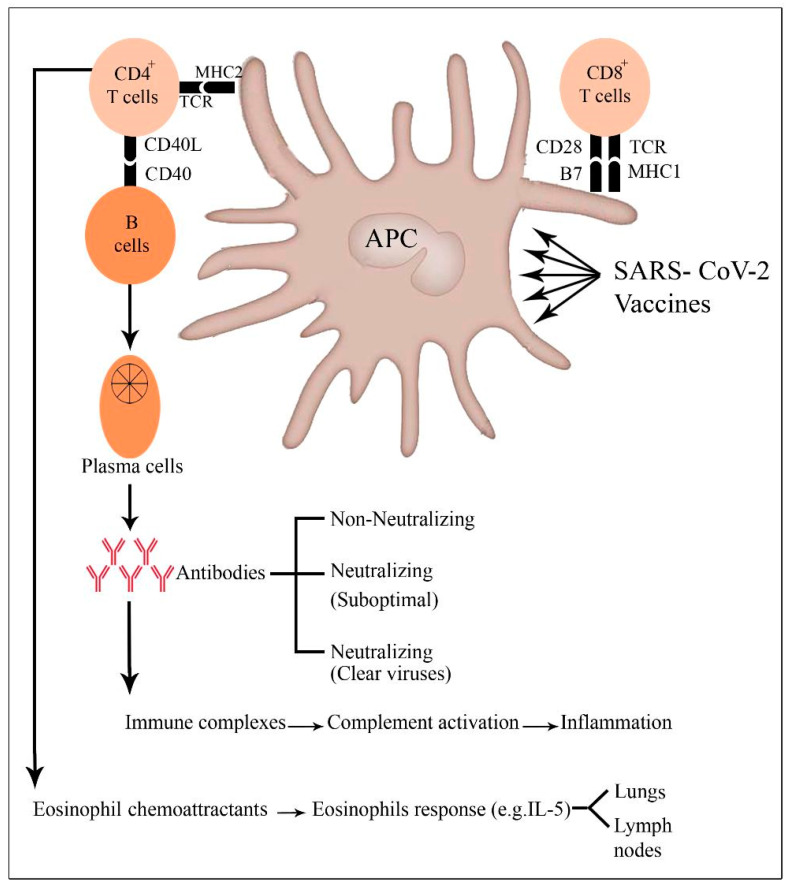
Immunological reactions during SARS-CoV-2 infection and after vaccination against COVID-19. APC, antigen-presenting cell; TCR, T cell receptor; MHC, major histocompatibility complex; L, ligand; CD, cluster of designation; IL, interleukin; B7, CD80 and CD86.

Immune responses that form quickly after infection resolution are not predictive of adult long-term memory [189]. This is because 28% to 50% of people not previously exposed to SARS-CoV-2 infection show cross-reactive T cells that were likely produced in response to other members of the coronavirus family, such as the common cold coronaviruses HCoV-OC43, HCoV-HKU1, and HCoV-229E [190]. Children may develop cross-protective antibodies against SARS-CoV-2 from typically infecting seasonal coronaviruses (Figure 4) [174,178,179,180].

#### 4.3.4. Cytokine Storm

In adults with severe and life-threatening COVID-19, the hyperinflammatory response (i.e., cytokine storm) begins in the second week of infection. Patients can suffer from acute respiratory distress syndrome (ARDS), multiorgan failure, and disseminated intravascular coagulation (DIC) due to the presence of elevated levels of circulating proinflammatory cytokines [191,192]. In ARDS, lung infiltration by macrophages and neutrophils initiates the cytokine storm [191,192]. These innate immunity cells generate potent inflammatory cytokines, such as IL-6, IL-12, IL-10, and TNF-α. Other studies have demonstrated elevated levels of IL-1, IL-7, IL-8, IL-17, and granulocyte-colony stimulating factor (G-CSF) [193,194]. The elevated levels of IL-6, IL-10, and C-reactive protein (CRP) are regarded as prognostic indicators [193,194]. In addition, lymphopenia, elevated ferritin, and D-dimer levels correlate with disease severity in adults infected with COVID-19 [192].

The profiles of cytokines produced by children and adults infected with SARS-CoV-2 vary [177,184,190,194]. The majority of them are essentially unaltered in severe/PIMS-TS, and there are moderate instances in children relative to their elevated levels in adults, despite their consistency in asymptomatic cases (Table 6).

**Table 5:** Key components during SARS-CoV-2 infection of the (**A**) B lymphocyte population, (**B**) T lymphocyte population, and (**C**) innate cell population.

(A) [67,173,174,176,195]						
Disease Severity	B Cells	Plasma Cells	IgG/IgA	B Cells	Plasma Cells	IgG/IgA
	(Adults)			(Children)		
Severe/PIMS-Ts	↓	↑	↑ ss	↓	↓	↓
Mild	uc	↑	↑ tr	uc	↑	↑
Asymptomatic	uc	↑	↑ tr	uc	↑	↑
**(B)** [174,176,182,195]						
**Disease Severity**	**CD3+**	**CD8+**	**CD4+**	**CD8+**	**CD8+**	**CD4+**
	**(Adults)**			**(Children)**		
Severe/PIMS-Ts	↓	↓	↓	↓	↓	↓
Mild	↑	↑	↑	↑	↑	↑
Asymptomatic	uc	uc	uc	uc	un	un
**(C)** [17,67,156,173,195]						
**Disease Severity**	**Monocytes**	**Neutrophils**	**NK**	**Monocytes**	**Neutrophils**	**NK**
	**(Adults)**			**(Children)**		
Severe/PIMS-Ts	↑	↑	↓	↓	↓	↓
Mild	↑	↑	↓	nr	nr	↑
Asymptomatic	nr	nr	↑	nr	nr	↑

**↑**, increased; **↓**, decreased; **ss**, sustained; tr, transient; uc, unchanged; nr, normal.

#### 4.3.5. PIMS-TS in Severe Pediatric COVID19

Cases in children resembling Kawasaki illness, a postinfectious inflammation of the medium-sized arteries, such as the coronaries, were identified in April 2020 [196,197,198,199]. Compared with prior years, the occurrence of these instances increased dramatically. Immunological investigations indicated a hyperinflammatory condition, distinct from both Kawasaki illness and the cytokine storm observed in severe, acute COVID-19 [200,201]. The condition is currently referred to as multisystem inflammatory syndrome in children, MIS-C (and in adults, MIS-A by the US Centers for Disease Control (CDC) and the WHO, or pediatric inflammatory multisystem syndrome temporally associated with SARS-CoV-2 (PIMS-TS) by the UK Royal College of Paediatrics and Child Health (RCPCH)). This is an uncommon condition that develops in children and young adults 1–2 months after a SARS-CoV-2 infection and is frequently asymptomatic or paucisymptomatic. This illness resembles toxic shock syndrome and is treated with corticosteroids and immunoglobulins [202]. In certain instances, immunomodulators, such as IL-1RA and anti-TNF, are used. The syndrome is characterized by autoantibodies that target diverse tissue antigens [200,203,204] in a protean fashion [205].

To explain the widespread immunological activation and dysregulation, it has been shown that a superantigen region in the SARS-CoV-2 spike protein has the capacity to bind, cross-link and activate T cells with specific T cell receptors (TCRs), resulting in the formation of chains [202,206,207,208,209]. In order to induce MIS-C, other pathogens bearing superantigen components may be required in addition to SARS-CoV-2 infection. These pathogens are likely restricted to children and adolescents. This theory might explain why MIS-C is uncommon among the elderly. Alternately, the initial immune response elicited by SARS-CoV-2 differs between children and adults (see above, Section 1), hence generating the required conditions for later superantigen-mediated MIS-C in just these individuals to enhance the immune response. This is further reinforced by the fact that MIS-C presents later than other superantigen-mediated illnesses.

In addition, MIS-C may require a genetic component to develop [210], including the presence of HLA-class I genes [209]. The gut, rather than the airway mucosa, is the source of superantigens in MIS-C [211], which provides another explanation for the delayed development of MIS-C. Possible explanations include extended viral shedding in the intestines of children, potentially beyond that of adults, and disturbance of the integrity of the intestinal barrier in MIS-C patients [211]. This is corroborated by the fact that the majority of children with MIS-C have intestinal symptoms and terminal ileum inflammation [212]. In genetically susceptible people, the SARS-CoV-2 virus can cause local superantigen-mediated T cell activation and inflammation, a loss of intestinal barrier integrity, and the release of superantigens into the bloodstream in children more so than in adults. These variables may account for the systemic hyperinflammatory responses observed in children and adolescents with MIS-C. Two-thirds of children with severe COVID-19 disease are affected by PIMS-TS [212]. The latter syndrome is characterized by a broad spectrum of clinical symptoms and varying disease severity, including shock, multiorgan failure, left ventricular dysfunction, and coronary artery anomalies [213]. Sometimes, the underlying cytokine storm in PIMS-TS is referred to as macrophage activation syndrome (MAS) [184,213].

Moreover, it was revealed that children with PIMS-TS have higher levels of IL-6, IFN-, TNF-, and CXCL10 (chemokine “C-X-C motif” ligand) than children with acute COVID-19 [186]. Adults with COVID-19 infection have abundant amounts of the chemokines CXCL10 (also known as Interferon gamma-induced protein 10), CXCL8, and CCL2 (CC chemokine ligand, also known as monocyte chemoattractant protein-1) [194]. Other studies, however, have demonstrated that the levels of IL-6, IFN-, and TNF in pediatric COVID-19 were stable and unchanged [181]. Similar to patients with severe adult COVID-19, patients with PIMS-TS showed high CRP, ferritin, and D-dimer values, indicating the inflammatory nature of the underlying disease [214]. These findings reveal that the immune responses and biomarker levels of people with severe COVID-19 and PIMS-TS are substantially comparable. In fact, the organ damage caused by these disorders is fairly unique. PIMS-TS does not appear to be a direct consequence of SARS-CoV-2 infection, but rather, a consequence of immunological alterations generated by SARS-CoV-2 in the host [215]. PIMS-TS patients arrived weeks after the peak of the first wave of COVID-19, and the majority of presenting children were SARS-CoV-2 PCR-negative and IgG-positive [213,214]. In contrast, adult COVID-19 patients may remain PCR-positive as the disease advances

### 4.4. Energy Allocation Perspective on COVID-19 in Children

Physical development is a significant physiological difference between children and adults. Humans are born underdeveloped and helpless; therefore, there is a strong evolutionary need to encourage early growth [216]. Consequently, energy allocation balances the necessity between immune responses and development, favoring the latter until the pathogen poses a grave threat to the child’s life and fitness [216]. Disease tolerance is an immune defense mechanism that is employed when the immune response to a pathogen is more harmful than the infection itself [216]. The decision to fight or tolerate a particular virus is likely to be different in a developing youngster than in an adult. It is hypothesized that adolescents are more prone to the adoption of illness tolerance to minimize the systemic inflammatory response whenever feasible. The energy needs of children between the ages of 0–20 years show a continuous decline, with boys requiring somewhat more energy than girls. Among all immune system processes, the systemic inflammatory response is the most expensive and frequently causes weight loss in children during viral episodes characterized by fever, muscular pain, and other symptoms of systemic inflammation. During SARS-CoV-2 infection, the greatest incidences of asymptomatic COVID-19 are observed in children with the greatest energy demands [217].

Moderate and asymptomatic COVID-19 infection in children is linked with seroconversion, the generation of neutralizing antibodies and specific T cells, but not with systemic inflammation or bystander T cell activation. Given that T cell-mediated responses are crucial for viral clearance, it is fair to assume that lower bystander T cell activation and a milder systemic inflammatory response may increase the chance of viral persistence. A recent report indicated that asymptomatic children infected with SARS-CoV-2 have similar adaptive T and/or B cell responses but lower cytokine responses [218].

If children and adolescents are more likely to select illness tolerance over disease resistance due to energy allocation for growth, this may explain why MIS-C is most prevalent in children and adolescents. Throughout childhood, boys have slightly greater energy needs than girls, which, according to the energy allocation hypothesis described herein, should translate to a greater predisposition for disease tolerance and more frequent mild or asymptomatic illnesses in boys [202,210].

## 5. Concluding Immunological Remarks

Infection with SARS-CoV-2 in children and the elderly manifests differently, depending mostly on the immunological response. Understanding the particular characteristics of the immune systems of children and adolescents can aid in the design of effective and efficient means of disease control, prevention, and therapy. It may also be possible to better comprehend other diseases involving the immune system, despite their varying symptoms over the age range. Since SARS-CoV-2 is a novel zoonotic pathogen, there is no pre-existing immunity; hence, the entire human population is susceptible to infection and the development of COVID-19 disease. All children are susceptible to SARS-CoV-2 infection, but the vast majority of pediatric cases are mild; serious COVID-19 disease is uncommon in children compared to adults [219]. Children’s low ACE2 receptor expression may contribute to their relative resistance [13,14,88,89,90,91,92]. However, the innate immune system of children may be the key to understanding SARS-CoV-2 resistance and vulnerability. In addition, the repeated infections that occur during the first few years of life contribute to the establishment of memory T and B cells that inhibit reinfection or disease development from commonly encountered pathogens [220].

In the majority of cases of COVID-19, the viral load peaks within the first week of infection, and patients generate their main immune response between days 10 and 14, followed by virus clearance, which is mediated by high-affinity antibodies and T cells. The response of naïve B cells to every fresh infection or vaccine occurs via the germinal center reaction and takes two weeks [221]. Children’s immune systems are equipped to respond to any disease, including SARS-CoV-2, based on three factors. First, natural antibodies play a crucial role in the early stages of an infection [222]. These antibodies are predominantly of the IgM isotype, produced independently of previous antigen exposure, and have a wide range of reactivity and a variable affinity. They can confine the infection for the two weeks required to produce high-affinity antibodies and memory B cells (MBCs), which eliminates the virus and prevents reinfection [223]. The switched MBCs produce high-affinity antibodies [223].

Second, youngsters are able to rapidly create natural antibodies with broad reactivity that have not yet been selected and modified by the immune response to prevalent environmental infections. This can be accomplished via the cooperation of CD27dim and CD27bright MBCs to produce antigen-specific antibodies or IgM (innate) antibodies, respectively [224].

Third, in infants and children, the majority of MBCs are CD27dull, making them highly adaptive to novel antigens. In contrast, in the elderly, the majority of MBCs are CD27bright and are incapable of adapting to novel antigens. Cytokine secretion is another function of B cells. Neonatal B cells, activated B cells, and IgA plasmablasts release the powerful anti-inflammatory cytokine IL-10 [225]. Consequently, the immunological response in children may have the dual purpose of providing protection and lowering immune-mediated tissue damage, particularly in the lungs.

## Figures and Tables

**Table 1:** Innate immunity and adaptive immunity during the neonatal and infancy periods in comparison to in childhood and young adulthood.

Neonates and Infants	Children to Young Adults	Information
Innate Immunity	Innate Immunity	References
Poor neutrophil functions and muted innate immunity at birth	More mature immunity (fewer infections and effective vaccination)	[21,22,23]
Reduced M/M functions	Waned maternal Ig	[24,25,26,27]
Reduced bioactive molecules and tissue repair	Normal bioactive molecule production and tissue repair	[28]
Fewer mDCs	Normal mDcs	[29]
Reduced PcDcs (limited α/β interferon)	Normal PcDcs	[30]
Low concentration of IL-12	Normal IL-12	[31]
Normal TLR4, -7, and -9	Normal LTR receptors	[30]
Reduced NK activity (50% of adult level)	Increased NK activity	[32]
Diminished concentration of alternative/lectin-binding complement pathways	Normal alternative/lectin-binding complementary pathways	[33]
**Adaptive Immunity**	**Adaptive Immunity**	
Maintained fetal Treg cells (self-tolerance)	Decreased Treg activity	[34,35,36,37]
Predominant Th2 response	Strong CD4 and CD8 activity	[38]
Enhanced γδ T cells (increased γ interferon production)	Increased CD45R naïve T cells	[39,40]
B1 cell stimulation (increased interferon production)	B cell hypermutation and nonexposure cross-reactions	[41,42]
B2 cell stimulation (IgM production) and short-lived plasma cells and Ig	Increased Ig production, increased plasma cells in the BM and retained antigens at the follicular DC	[41,43,44]
Diminished Th1 and CD8 activity, enhanced T17 activity	Increased Th1, Th2, and Th17 numbers and activity	[45,46,47,48]
Limited generation of B and T memory cells (limited hypermutation)	Generation of B and T memory cells, effective vaccination	[49,50,51,52,53,54,55,56,57,58]
Limited involvement of the classical complement pathway (low CD21)	Increased classical complementary pathway activity	[59]

M/M, monocyte–macrophage; Ig, immunoglobulin; mDC, myeloid dendritic cell; PcDC, plasmacytoid dendritic cell; TLR, Toll-like receptor; NK, natural killer cell; Treg, T regulatory cell; BM, bone marrow; CD, cluster of designation; DC, dendritic cell. The other type of dendritic cells (plasmacytoid DC, pDC) produce compromised levels of interferon alpha and beta upon viral exposure in spite of expressing the usual adult levels of LTR7 and LTR9 [30].

**Table 3:** Key components of innate immunity against SARS-CoV-2 infection.

Immunocompetent Cells	Immunological Elements	Functions
Dendritic cells (DCs)	Plasmacytoid (pDC) and myeloid (mDC)	- Antigen-presenting cells
		- mDC links innate immunity with adaptive immunity
		- pDC produces INF-α and INF-β upon viral exposure and expresses LTR7/9 [138,140]
Neutrophils	Phagocytic cells	- Neutrophil activation is lower in pediatric COVID-19 [141]
Monocyte–macrophages	Phagocytic cells	- Antigen-presenting cells to T cells
		- Recruitment of lymphocytes (elements of
		adaptive immunity)
		- Production of varieties of bioactive molecules
		- Activation of monocyte–macrophages enhanced via trained immunity [138,139,140]
Natural killer (NK) cell	Granzymes, perforin, IFN-γ, TNF-α, and FASL expression	- Cytolytic granule-mediated cell apoptosis
		- Antibody-dependent cell-mediated cytotoxicity (ADCC)
		- Cytokine-induced NK and cytotoxic T lymphocyte (CTL) activation
		- Killing of virally infected cells (missing “self” hypothesis)
		- Brief decrease in NK cells after the onset of COVID-19 symptoms [138,140]
Natural antibodies	Antibodies produced before viral infection or immunization	- Activation of the classical complement pathway
		- Antimicrobial activities [95,96]
Alternative (properdin) complement pathway	C3 to C9 and other factors	- Part of the innate immunity
		- Does not require antibodies for activation [95,96]
Epithelial and mucous membrane barriers	Mechanical barrier with protective armory	- Prevents microbial entrance
		- Virus must attach to it before entrance [95,138]

**Table 6:** Comparison between the cytokine profiles of the different clinical categories of COVID-19 among children and adults (cited from [195], Open Access Journal).

Adults			Children			
Severe	Mild	Asymptomatic	PIMS-TS	Severe	Mild	Asymptomatic
↑ IL-6	↑ IL-6	↔ IL-6	↑ IL-6	↔ IL-6	↔ IL-6	↔ IL-6
↑ IL-10	↑ IL-10	↑ IL-10	↑ IL-10	↑ IL-10	↑ IL-10	↑ IL-10
↑ IFN	↑ IFN	↔ IFN	↑ IFN	↑/↔ IFN	↑/↔ IFN	↔ IFN
↑ TNF-α	↑ TNF-α	↔ TNF-α	↑ TNF-α	↔ TNF-α	↔ TNF-α	↔ TNF-α
↑/↔ IL-1β	↑/↔ IL-1β	↔ IL-1β	n/a	↔ IL-1β	↔ IL-1β	↔ IL-1β
↑/↔ IL-8	↑/↔ IL-8	↔ IL-8	↑ IL-8	↑ IL-8	↔ IL-8	↔ IL-8
↑/↔ IL-17	↑/↔ IL-17	↔ IL-17	↑ IL-17	↑/↔ IL-17	↑/↔ IL-17	↔ IL-17

↑, increased; ↔, unchanged; IFN, interferon; IL, interleukin; n/a, not available; TNF-α, tumor necrosis factor alpha.

## Data Availability

Not applicable.
